# Over 30 years of clinical reasoning: lessons from the “case records”

**DOI:** 10.3389/fmed.2025.1691085

**Published:** 2025-10-29

**Authors:** Mohammed Abdulrasak

**Affiliations:** ^1^Department of Clinical Sciences, Lund University, Malmö, Sweden; ^2^Department of Gastroenterology and Nutrition, Skåne University Hospital, Malmö, Sweden

**Keywords:** medical education, clinical conferences, grand rounds, diagnostic reasoning, thought process

For reasons I still can't fully explain, I spent several years during my residency training reading over three decades of *Case records of the Massachusetts general hospital*, from 1990 onwards. The decision was neither part of a research project nor assigned coursework—it began as curiosity and slowly turned into ritual. I read them the way some read novels or memoirs: with attention to character, context, tone, and suspense. And over time, something remarkable happened. I wasn't just learning medicine; *I was learning how medicine had changed over time*.

These case records—clinical-pathologic conferences (CPCs) dating back more than a century—are unique artifacts in medical literature. Part diagnosis, part detective story, part philosophical exercise, they ask us to reason with incomplete information, to entertain wrong paths, and to watch a diagnosis unfold in slow, deliberate layers. Over 30 years of cases, I noticed not only the arc of countless individual stories, but also the story of medicine itself: how it thinks, how it changes, and how it sometimes forgets what it once knew.

Reading the cases in chronological order gave me a strange advantage. I saw, in real time, how the diagnostic process adapted to new technologies, shifting norms, and global events. The 1990s often opened with detailed physical exams and long, hypothesis-driven narratives. In an era when MRIs were still novel and genetic testing rare, the diagnostic weight often fell on observation, pattern recognition, and clinical memory. Case discussions were dense with clinical pearls, eponymous syndromes, and nuanced inferences from the patient's history.

By the mid-2000s, the tone had shifted. Imaging played a more prominent role in the diagnostic process; molecular diagnostics began to appear. Diagnostic timelines shortened. Clinical narratives were still thoughtful, but the diagnostic toolbox had expanded, and the confidence of discussants often rose in proportion to available data. By the 2010s, next-generation sequencing, PET scans, and multidisciplinary tumor boards featured regularly. Some cases began to feel more like data integrations than mystery stories. The thrill remained, but its contours had changed.

Across the decades, the case records not only reflected changes in diagnostic tools, but also revealed how clinical reasoning itself evolved. In a 1990 case involving suspected tuberculosis–sarcoidosis overlap (Case 24-1990) (1), the discussants built their differential diagnosis through careful clinical pattern recognition and exclusion, relying on radiographic findings, clinical memory, and structured inference. In contrast, Case 23-2010 presented a yet-unnamed constellation of findings—telangiectasias, erythrocytosis, monoclonal gammopathy, perinephric fluid collections, and intrapulmonary shunting—that would later be identified as TEMPI syndrome (2, 3). At the time of presentation, the diagnosis did not yet exist in medical literature. The discussants nevertheless formed a coherent clinical narrative by synthesizing disparate data which led to the formation of a newly identified syndrome a short time later (3). Together, these cases illustrate the shift from a tactile, analog style of reasoning to a more integrative, data-assisted approach.

Another noticeable evolution was in the endpoint of diagnostic certainty. In earlier decades, the diagnosis often emerged from the autopsy table—a final confirmation that resisted dispute. These were cases of retrospective revelation, where the truth was fixed in tissue. Over time, however, autopsy became less central. Diagnoses were increasingly made during the life of the patient at hand, based on imaging, endoscopy, biopsy, or molecular testing. The shift marked a subtle but profound change: a move from definitive post-mortem truth to a living, probabilistic form of knowing. While it reflects remarkable diagnostic progress, it also raises the question of how we define certainty now.

Over time, there was a growing sensitivity to the language of care. The terms “patient-centered” and “shared decision-making” began to appear with increasing frequency (4). In more recent cases, the perspective of the patient's family—how relatives interpreted, contributed to, or were affected by the clinical picture—was sometimes included, underscoring the “familial” nature of illness (5). There was also a marked shift toward humility—an openness about uncertainty, especially in cases involving complex, multisystem disease. This evolution in tone was not just semantics; it reflected a deeper transformation in how clinicians relate to patients, and how they think about what constitutes care.

What remained constant, though, was the pedagogical power of these conferences. At their best, they are not about getting the diagnosis right. They are about showing how to think and how to ask the right questions, how to tolerate ambiguity, how to delay closure. They model cognitive flexibility—watching seasoned physicians revise hypotheses, change direction, and admit knowledge gaps in real time. In today's world of instant answers, such slow thinking feels almost radical.

One case that left a lasting impression involved a 40-year-old woman who presented with abdominal pain, nausea, weight loss, and a consuming fear that she had pancreatic cancer (Case 33-2013) (6). Despite repeated normal investigations and reassurance, her conviction persisted—driven by health-related anxiety and sustained by extensive online research. Ultimately, she was diagnosed with somatic symptom disorder, and the focus of her care shifted from ruling out rare disease to managing the psychological burden of uncertainty. The case was not memorable because of a dramatic diagnosis, but because of how it revealed the human cost of diagnostic overreach—and the ethical challenge of caring without over-testing. The discussants modeled a different kind of clinical wisdom: one grounded in restraint, empathy, and respect for the patient's narrative.

As artificial intelligence becomes increasingly utilized in diagnostic medicine, there is a temptation to relegate this kind of slow, reflective reasoning to the past. But rather than replace the CPC model, AI could—and should—complement it. Imagine a case discussion where an AI system offers differential probabilities based on structured data, while clinicians push back with context, exceptions, and stories. CPCs could become testing grounds for this kind of collaborative cognition: not man vs. machine, but man with machine, grappling together with clinical complexity. The case records, then, are not obsolete—they are fertile ground for understanding where human judgment must still lead.

Reading the case records was, in a way, a longitudinal study in humility. I began expecting to learn about diseases. I ended up learning about thinking. I saw how the best clinicians resist the urge to be certain, how they interrogate their own assumptions, how they return—again and again—to the patient's story when the data seems unclear. And yet, along the way, I also expanded my clinical armamentarium. With each case, I gained diagnostic strategies and frameworks that have become embedded in how I now practice medicine—lessons that continue to shape how I encounter complexity, even years later.

Although I could rarely recall the exact details of individual cases in the majority of times, I found that certain diagnostic patterns were entrenched in the memory. These impressions surfaced unbidden during clinical encounters, guiding my thinking in ways I couldn't always trace. The case records didn't furnish me with perfect retention, but they gave me something subtler: a form of pattern memory. This, perhaps more than specific knowledge, is what makes them enduringly useful.

These are lessons worth preserving. If we want future physicians to be more than technicians—to be thinkers, stewards, and storytellers—we need to defend the spaces that teach those skills. CPCs, especially those as rigorous and reflective as the case records, are one such space. They model a form of intellectual integrity that is both timeless and urgently needed.

I do not know whether CPCs will persist in the same form for the next 30 years. But I hope they do. Or at the very least, I hope we continue to teach clinical reasoning not as an outcome, but as a craft. Reading the case records taught me that even in an era of limitless information, the patient's story still holds the final clue—if we are willing to listen slowly enough to hear it. This part of medicine has not changed over time, and it's hard to imagine that it ever truly will.
